# Comparison of respiratory muscle activities and cardiovascular function during different breathing types in healthy adults: Costal breathing, flow incentive spirometry, and volume incentive spirometry

**DOI:** 10.1371/journal.pone.0328739

**Published:** 2025-07-28

**Authors:** Preeyaphorn Songsorn, Kanyapit Chummee, Chutima Yaanan, Anurak Banlueharn, Kornanong Yuenyongchaiwat, Chalearmpong Pinupong, Sasipa Buranapuntalug

**Affiliations:** 1 Department of Physical Therapy, Faculty of Allied Health Sciences, Thammasat University, Pathumthani, Thailand; 2 Thammasat University Research Unit for Physical Therapy in Respiratory and Cardiovascular Systems, Thammasat University, Pathumthani, Thailand; Ordu University, TÜRKIYE

## Abstract

**Background:**

Various breathing exercises are used to enhance lung function. However, many patients have reported experiencing breathlessness and an increased work of breathing during their use, but evidence supporting this is limited. This study aimed to compare respiratory muscle activity and cardiovascular function during different breathing exercises.

**Methods:**

Forty-five healthy adults participated in this study. They were randomly assigned in a crossover design involving three breathing techniques: flow incentive spirometry, volume incentive spirometry, and costal breathing exercises with sustained maximal inspiration. Respiratory muscle activity (sternocleidomastoid, superior external intercostal, and inferior external intercostal) and cardiovascular function were assessed during each technique at baseline and during the 3^rd^, 6^th^, and 9^th^ breaths.

**Results:**

Sternocleidomastoid, superior external intercostal, and inferior external intercostal were more activated during flow incentive spirometry and volume incentive spirometry than during costal breathing exercises with sustained maximal inspiration. During flow incentive spirometry, stroke volume and heart rate significantly increased, while cardiac output significantly decreased compared to volume incentive spirometry and costal breathing exercises with sustained maximal inspiration throughout the 10 breathing cycles.

**Conclusion:**

Flow incentive spirometry and volume incentive spirometry elicited greater accessory muscle activity than costal breathing exercises with sustained maximal inspiration. Inferior external intercostal was activated in all breathing techniques. In particular, flow incentive spirometry stimulated changes in cardiovascular function more than volume incentive spirometry and costal breathing exercises with sustained maximal inspiration. Therefore, volume incentive spirometry and costal breathing exercises with sustained maximal inspiration are recommended to minimize breathing effort and cardiovascular instability.

## Introduction

Pulmonary disease is one of the four main diseases that increased the patient mortality rate from 1990 to 2021 [1]. Moreover, this disease can deteriorate the lungs and airways, affecting impaired quality of life and can lead to future disability. The main problem in pulmonary diseases is impaired pulmonary function, including decreased lung volume, and impaired gas exchange [2]. These problems can be addressed using medications and physical therapy to improve pulmonary function and prevent pulmonary complications.

Pulmonary physiotherapy aims to reduce breathlessness, decrease the work of breathing, enhance airway clearance, increase lung volume, improve gas exchange, and prevent or reduce post-pulmonary complications [3]. Several physical therapy methods aim at increasing lung volume and improving gas exchange. Commonly used techniques include diaphragmatic breathing exercises, costal breathing exercises (costal BE), and sustained maximal inspiration (SMI). Equipment-based breathing exercises commonly employed in hospitals and at home include flow-controlled incentive spirometry (FIS), volume-controlled incentive spirometry (VIS), and resistance breathing training. These devices are commonly utilized as they provide immediate feedback on the effectiveness of breathing exercises.

Costal BE combined with SMI (CSMI) increases lung volume, improves ventilation, and enhances gas exchange. This training focuses on specific lung lobes and may stimulate diaphragmatic muscle activity, especially when targeting the lower lobes, leading to greater lung expansion and improved gas exchange through breath retention [3]. The breathing pattern in CSMI is similar to that in breathing exercises using devices such as the FIS and VIS. During these exercises, the goal is to fully inhale and keep the ball or indicator in the device elevated for as long as possible [4]. The FIS is based on the principle of flow dependence, whereas the VIS is volume-dependent. Both CSMI and training with the FIS and VIS involve similar breath retention techniques.

FIS and VIS are commonly used and recommended in clinical practice to improve pulmonary ventilation or increase chest wall expansion. Previous studies assessing respiratory muscle activity using surface electromyography after thoracic surgery found that FIS stimulated more activity in accessory breathing muscles, potentially leading to adverse effects such as breathlessness and fatigue in the respiratory and accessory muscles [4]. In contrast to FIS, VIS is associated with reduced activation of accessory respiratory muscles. This decreased muscular engagement may contribute to a diminished perception of breathlessness and lower levels of muscle fatigue. Furthermore, VIS promotes deep inhalation accompanied by breath retention, which significantly increases alveolar distending pressure and prolongs negative pleural pressure, thereby elevating intrathoracic pressure. Although this rise in thoracic pressure has the potential to influence cardiovascular function, conclusive evidence from previous studies remains limited.

This study aimed to compare respiratory muscle activities and cardiovascular functions during different types of breathing exercises, including flow incentive spirometry (FIS), volume incentive spirometry (VIS), and costal breathing (CSMI), in healthy adults.

## Materials and methods

### Study design and sample

This study was designed as a randomized crossover trial of the order of breathing techniques (FIS, VIS, and CSMI). All participants provided informed consent in both written and verbal forms before their inclusion in the study, in accordance with ethical guidelines, and the study was approved by Human Research Ethics Committee of Thammasat University (Sciences) under approval number 070/2566. The study protocol was registered Thai Clinical Trials Registry (TCTR20240609002).

This study recruited 45 voluntary participants. The sample size was determined based on an effect size of 0.25, a statistical power of 80%, and a significance level of 0.05. Considering the three groups, four measurement points, and an anticipated dropout rate of 10%, the total required number of participants was calculated to be 45. The recruitment period started on June 5, 2024, and end on July 27, 2024.

The inclusion criteria were as follows: aged 25 to 50 years [5], body mass index (BMI) of 18.5 to 22.9 kg/m^2^ for Asian populations [6] and normal spirometry with a forced vital capacity >80% predicted, forced expiratory volume in one second >80% predicted and ratio of forced expiratory volume in one second and forced vital capacity > 0.7 [7]. Participants were excluded if they had cardiac or pulmonary symptoms, including chest pain, breathlessness at rest, orthopnea, a physician-diagnosed cardiac disease, or pulmonary disease, or musculoskeletal chest wall conditions such as scoliosis of the upper-middle thoracic and fracture of the chest wall, and high blood pressure of > 140/90 mmHg [8].

### Procedure

**Fig 1:**
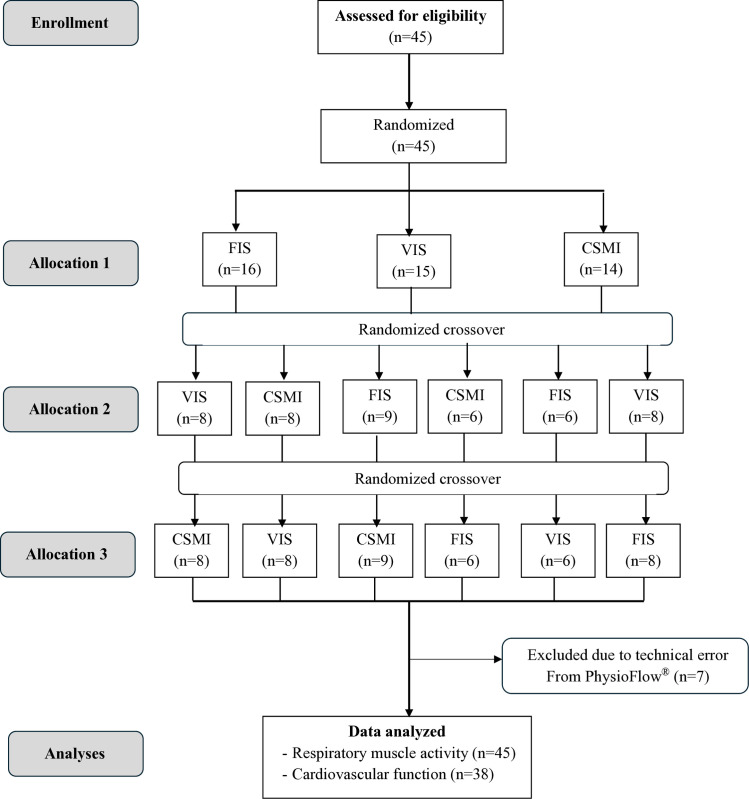
Consort flow diagram of the participants.

Participants were recruited according to the inclusion and exclusion criteria. All participants were asked to avoid coffee or tea for at least 4 hours before testing to avoid the effect of Caffeine on cardiovascular function effect such as heart rate or blood pressure [9]. The three breathing techniques were randomly assigned in the order of FIS, VIS, and CSMI (Fig 1). Wireless surface electromyography and PhysioFlow^®^ were conducted to measure the respiratory muscle activity and cardiovascular function at baseline and during breathing at 3^rd^, 6^th^, and 9^th^ breathing cycle.

All participants were randomly assigned to a sequence of breathing exercises. They were instructed to sit upright in a comfortable, relaxed position which was maintained throughout the test. A pillow was placed on each participant’s right side to help relax all muscles on that side. Each breathing technique was performed for 10 breathing cycles at a resting pace [10,11]. All participants rested between breathing techniques for 5 min or until respiratory rate, heart rate, and all respiratory muscle activities recovered to the baseline to eliminate cumulative effects from previous breathing techniques. The same research assistant taught all three breathing techniques to avoid differences in instruction [12].

During CSMI, the research assistant placed their hands on the lower part of the chest wall (below the inferior angles of the scapula on both sides). Participants were then instructed to breathe in slowly and fully to maximal inspiration through the nostrils until reaching total lung capacity, hold their breath for 3 s, and breathe out via their mouth [12].

FIS and VIS were performed using Triflo II and Voldyne^®^ 4000, respectively. The participant held the FIS or VIS at eye level on the left-hand side. They were asked to breathe in via the mouthpiece of the FIS or VIS until reaching total lung capacity, hold their breath for 3 s using visual feedback from the balls and plate, and then breathe out [10,11,13]. During the VIS, the participants were instructed to breathe, raising the plate in the volume chamber as much as possible while sustaining the plate in the flow chamber at the smiling face symbol indicator, then maintained for 3 s. For the FIS, participants were instructed to raise the three balls as high as possible and maintain them for 3 s [12].

### Study location

The study was conducted in Room 302, 3rd Floor, Piyachart Building, Thammasat University (Rangsit Campus), Thailand.

### Respiratory muscle activity

A wireless surface electromyography was used to measure respiratory muscle activity. The skin area of the electrodes was cleaned with an abrasive gel and alcohol before placing the surface electrodes. Participants were instructed to sit in an upright position, and bipolar electrodes were placed bilaterally in three areas as follows: 1) Right sternocleidomastoid muscle; electrodes were placed on the middle 3rd of the muscle belly [14,15], with a reference electrode placed on the C5–C6 spinous process; 2) Right superior external intercostal muscle: electrodes were placed at the 2nd anterior intercostal space in the right midclavicular line of the chest wall [16] with the ground electrode placed on the right acromion process; and 3) Right inferior external intercostal muscle: electrodes were placed at the 7^th^–8^th^ anterior intercostal space in the right midclavicular line [16] with the reference electrode at the xiphoid process. Electromyography (EMG) signals were recorded at a frequency of 2000 Hz using an 8-channel wireless system, Ultium^®^ EMG (Noraxon^®^, Arizona, USA). These signals were filtered with a bandpass range of 20–500 Hz and processed using the root mean square method with a 300-millisecond window period. Respiratory muscle activity parameters included respiratory muscle activities from sternocleidomastoid muscle (SCM), superior external intercostal muscle (SEIM), and inferior external intercostal muscle (IEIM). All participants performed maximal voluntary contractions (MVC) of the inspiratory muscles for data normalization. They were instructed to sit upright in a comfortable, relaxed position during MVC test. During the test, participants were asked to inhale through a respiratory pressure meter, MicroRPM™ (Micro Medical/CareFusion, Kent, United Kingdom), using equipment to assess maximal inspiratory muscle strength [15]. One research assistant collected and instructed these variables.

### Cardiovascular function

This study used the noninvasive cardiac impedance method, PhysioFlow^®^ (Enduro^TM^ technology, Paris, France), to measure cardiovascular function. This method is based on the cardiac impedance principle, which is highly correlated with gold standard methods [17]. Six electrodes were placed as follows: (1) two on the left lateral aspect of the neck, (2) one in the middle of the sternum, (3) one in the mid-axillary line at the 5th intercostal space, and (4) two on the back at the level of the xiphoid process in which the changes in signals from intrathoracic hemodynamics were transferred via these electrodes. Cardiovascular function parameters included stroke volume (SV), heart rate (HR), and cardiac output (CO). Calibration using simultaneous inputs of blood pressure and electrocardiography signals as reference values was routinely performed to ensure the accuracy and reproducibility of these data. A trained research assistant collected these variables.

All participants were randomly assigned to a sequence of breathing exercises. The participants were asked to sit upright in a comfortable and relaxed position. Each participant’s right side was placed on a pillow to relax all muscles on that side. Each breathing technique was performed for 10 breathing cycles at a resting pace [10,11]. All participants rested between breathing techniques for 5 min or until respiratory rate, heart rate, and all respiratory muscle activities recovered to the baseline to eliminate cumulative effects from previous breathing techniques. The same research assistant taught all three breathing techniques to avoid differences in instruction [12].

During CSMI, the research assistant placed their hands on the lower part of the chest wall (below the inferior angles of the scapula on both sides). Participants were then instructed to breathe in slowly and fully to maximal inspiration through the nostrils until reaching total lung capacity, hold their breath for 3 s, and breathe out via their mouth [12].

FIS and VIS were performed using Triflo II and Voldyne^®^ 4000, respectively. The participant held the FIS or VIS at eye level on the left-hand side. They were asked to breathe in via the mouthpiece of the FIS or VIS until reaching total lung capacity, hold their breath for 3 s using visual feedback from the balls and plate, and then breathe out [10,11,13]. During the VIS, the participants were instructed to breathe, raising the plate in the volume chamber as much as possible while sustaining the plate in the flow chamber at the smiling face symbol indicator, then maintained for 3 s. For the FIS, participants were instructed to raise the three balls as high as possible and maintain them for 3 s [12].

### Statistical analysis

Data analysis was performed using SPSS software (version 22). Categorical variable was presented using frequency, while continuous variables were presented using mean and standard deviation (mean ± SD). Normality of the respiratory muscle activity and cardiovascular function variables were assessed using the Shapiro–Wilk test. Both the respiratory muscle activity and cardiovascular function variables were analyzed using two-way mixed repeated measures ANOVA with Bonferroni correction to compare within-group differences (baseline, 3^rd^, 6^th^, and 9^th^ breathing cycles) and differences between the three breathing techniques. Statistical significance was set at p-value less than 0.05 (*p* < 0.05).

## Results

Participants (n = 45) who met the inclusion and exclusion criteria were enrolled in this study (Table 1). A total of 45 healthy adults participated in this study. The mean age of the participants was 34.6 years (±7.8 years). The average body weight was 56.3 kg (±7.9 kg), and the mean height was 163.6 cm (±8.3 cm). The calculated mean BMI was 21.0 kg/m² (±1.4 kg/m²), which falls within the normal BMI as defined by the World Health Organization [6]. The gender distribution consisted of 17 males (7.78%) and 28 females (62.22%).

**Table 1:** Characteristics of all participants in this study.

Characteristics	Healthy adult (n = 45)
Age (years)	34.6 ± 7.8
Weight (kg)	56.3 ± 7.9
Height (m)	163.6 ± 8.3
BMI (kg/m^2^)	21.0 ± 1.4
Gender -Male -Female	17 (37.78) 28 (62.22)

Note: Data are presented as mean ± standard deviation or number (percentage).

### Respiratory muscle activity

**Fig 2:**
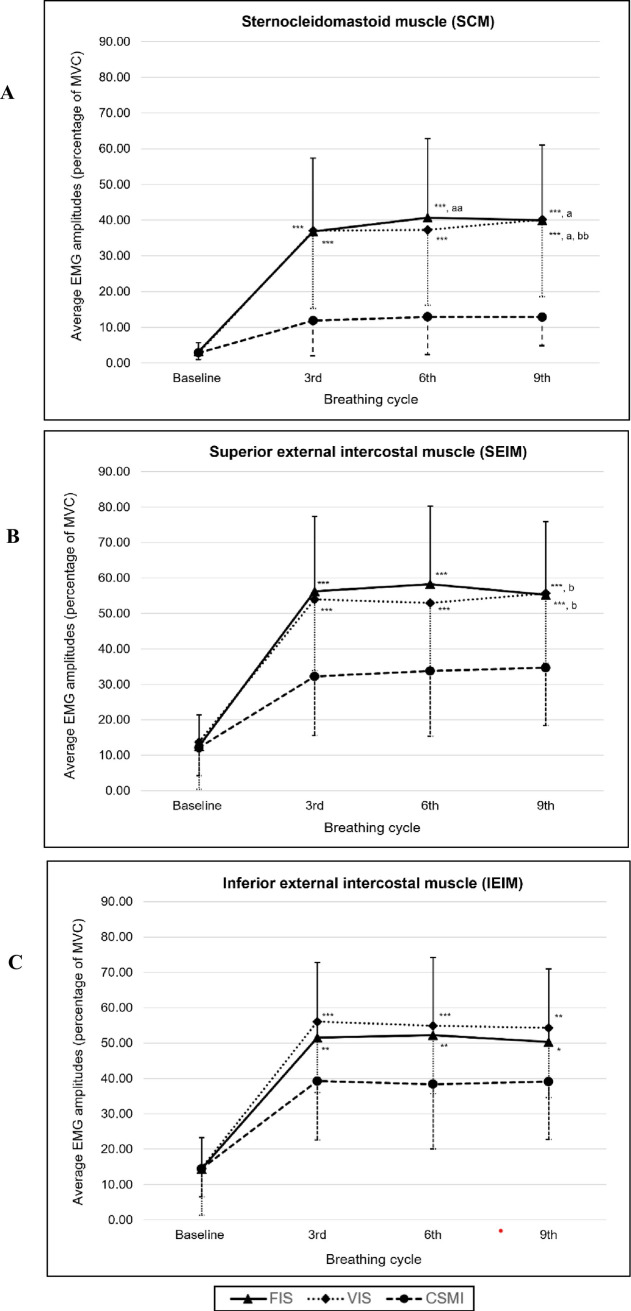
The comparison of respiratory muscle activities between FIS, VIS, and CSMI at baseline, 3^rd^, 6^th^, and 9^th^ breathing cycle; (A) sternocleidomastoid muscle, (B) superior external intercostal muscle, and (C) inferior external intercostal muscle. * *p* < 0.05, ** *p* < 0.01, ***p < 0.001 compared to CSMI, ^a^ *p* < 0.05, ^aa^ *p* < 0.01 compared to 3^rd^ breathing cycle, ^b^ *p* < 0.05, ^bb^ *p* < 0.05 compared to 6^th^ breathing cycle.

At baseline, none of the respiratory muscle activities differed between the types of breathing exercises (*p* > 0.05). SCM, SEIM, and IEIM activities increased significantly from baseline (*p* < 0.001) for all breathing techniques throughout 3^rd^, 6^th^, and 9^th^ breathing cycles. SCM, SEIM, and IEIM activities in the FIS and VIS were significantly higher than those in the CSMI during the 3^rd^, 6^th^, and 9^th^ breathing cycles (*p* < 0.001 for SCM, SEIM, and IEIM in FIS and VIS compared to CSMI at the 3rd, 6th, and 9th breathing cycles). However, no muscle activity differed significantly between the FIS and VIS groups during the 3^rd^, 6^th^, and 9^th^ breathing cycles (*p > *0.05) (Fig 2).

### Cardiovascular function

Seven participants were excluded from the cardiovascular function analysis due to a technical error with PhysioFlow^®^, leaving a total of 38 participants (n = 38) (Fig 1). CO, HR, and SV show no significant differences at baseline between the breathing techniques (*p > *0.05).

**Fig 3:**
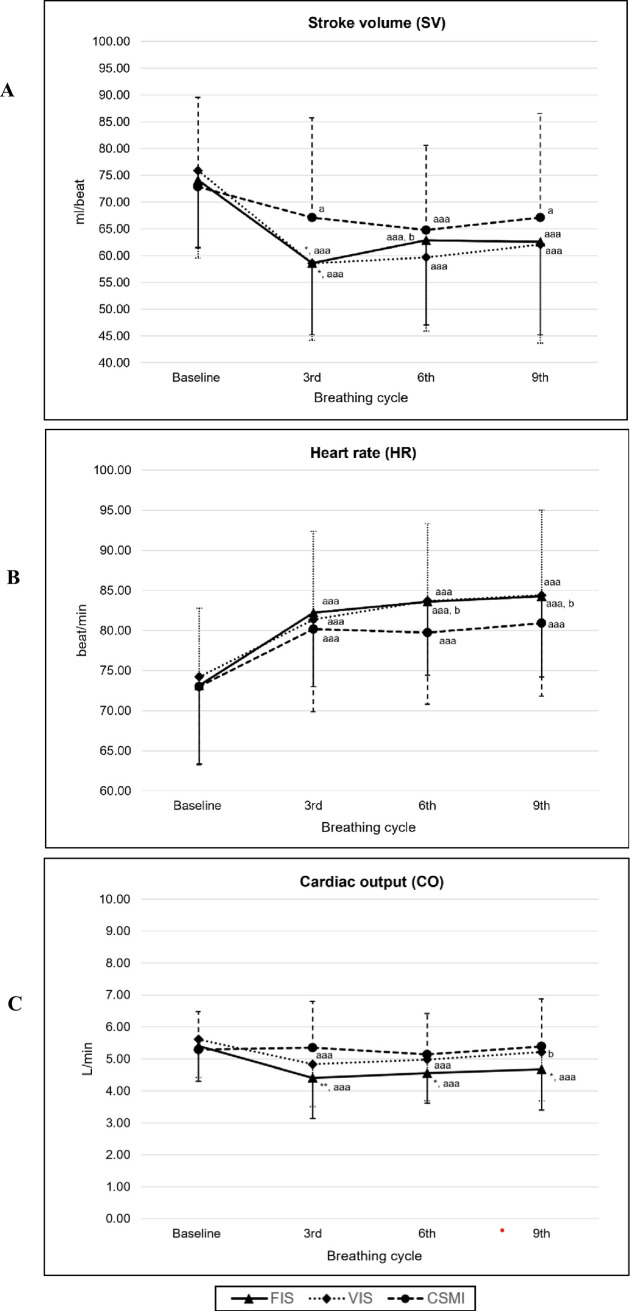
The comparison of the cardiovascular function variables at baseline, 3^rd^, 6^th^, and 9^th^ breathing cycle between three types of breathing exercises, presented as Mean ± SD (n = 38). * *p* < 0.05 compared to CSMI, ^a^ *p* < 0.05, ^aa^ *p* < 0.01, ^aaa^ *p* < 0.001 compared to baseline. ^b^ *p* < 0.05 compared to 3^rd^ breathing cycle.

While performing FIS, VIS, and CSMI, the SV significantly decreased at the 3^rd^, 6^th^, and 9^th^ breathing cycles from baseline (*p* < 0.001, all breathing techniques). SV during FIS and VIS was significantly lower than during CSMI at the 3^rd^ breathing cycle (FIS vs CSMI: *p = *0.020 and VIS vs CSMI: *p = *0.019) (Fig 3). However, the 6^th^ and 9^th^ breathing cycles did not show significantly different results between the three breathing techniques (*p > *0.05).

HR during FIS, VIS, and CSMI showed a significant increase at the 3^rd^, 6^th^, and 9^th^ breathing cycles from baseline (*p* < 0.001). A comparison of breathing techniques revealed no significant differences between VIS, FIS, and CSMI (*p > *0.05) (Fig 3).

At the 3^rd^, 6^th^, and 9^th^ breathing cycles, FIS and VIS showed a significant decrease in CO from baseline (*p* < 0.001). Conversely, CO during CSMI slightly increased, but there was no significant difference compared with baseline (*p* > 0.05). However, FIS showed a significant decrease in CO at 3^rd^ (p = 0.003), 6^th^ (p = 0.034), and 9^th^ (p = 0.033) when compared to CSMI (Fig 3).

## Discussion

### Respiratory muscle activity

Our results showed that SEIM and IEIM activities were slightly stimulated to move the rib cage outward following tidal volume breathing at rest. SCM was not stimulated during the resting period. These data confirmed that all participants were fully rested at baseline. SEIM and IEIM activities slightly increased to move the rib cage outward during resting tidal breathing, while SCM remained inactive, confirming that participants were fully rested at baseline.

The electrical muscle activity of the SCM (approximately +30% MVC) and SEIM (approximately +40% MVC) increased significantly in both FIS and VIS groups compared to baseline. The highest activation occurred during FIS, although no significant difference was found between FIS and VIS during performing breathing exercise. This suggests that both devices effectively stimulated accessory respiratory muscles due to the need to generate airflow and increase transpulmonary pressure to achieve maximal inspiration and maintain the position of the plate or balls of the spirometer [18]. Although VIS and FIS produced similar activation patterns overall, SCM and SEIM activity was the highest during FIS at the 3^rd^, 6^th^, and 9^th^ cycle. These findings align with previous research indicating. FIS requires a higher flow rate (approximately 0.7 L/s) than VIS (approximately 0.4 L/s) [12]. These differences are associated with the type of incentive spirometer, where VIS requires volume-dependent breathing, and FIS requires flow-dependent breathing. Therefore, to lift and sustain the three balls in FIS, participants had to generate greater airflow which required increased SCM and SEIM recruitment to enhance transpulmonary pressure [18–21]. This was related to increased loading in the respiratory system, which increased the work of breathing with FIS [12,15,22,23]. Interestingly, SEIM activity decreased at the 9^th^ breathing during FIS compared to the 6^th^ breathing cycle, but this was not observed with VIS. This reduction suggests a decrease in motor unit recruitment, potentially indicating that the use of FIS places greater demand on the muscle, leading to rapid energy depletion and a subsequent reduction in the number of motor units available for activation [24,25]. Therefore, the number of FIS repetitions should be considered, because excessive repetitions may contribute to muscle fatigue.

Our findings showed that IEIM activity was highest during VIS, although the difference between VIS and FIS was not statistically significant. This findings aligns with previous studies by Parreira et al. [18] and Paisani et al. [16], which reported that VIS promotes lower respiratory muscle activation and greater chest wall expansion, particularly through abdominal motion. This change occurred because of the low flow rate during VIS, which generated laminar flow and allowed air to redistribute to the peripheral areas [13,26]. This flow pattern enhances optimal diaphragmatic movement and improves chest wall expansion in the basal area, contributing to increased displacement of the abdominal compartment and reduced SCM and SEIM activities. These mechanisms are consistent with our finding that VIS stimulated SCM and SEIM activities less than FIS, although the difference was not statistically significant.

CSMI elicited lower SCM, SEIM, and IEIM activation compared to FIS and VIS. However, this technique maintained consistent motor unit recruitment across the 3^rd^ to 9^th^ breathing cycles in all three muscles. Although IEIM activity was not markedly elevated, CSMI induced less stimulation of SCM and SEIM than VIS and FIS. This technique targets lower-chest breathing, initiated by manual guidance from the research assistant and followed by a 3-second inspiratory hold at total lung capacity. IEIM activation in CSMI was likely due to the costal breathing component, which directs airflow toward the lower thoracic region [27]. Previous studies reported that intercostal and SMI breathing exercises increase chest wall volume more than diaphragmatic breathing or quiet breathing [27]. CSMI integrates both costal breathing, targeting specific lung regions, and SMI, a general lung expansion technique that incorporates a sustained inspiratory hold. Like VIS, CSMI promotes slow inspiratory flow and inspiratory hold, potentially facilitating collateral ventilation and airflow redistribute to peripheral airways [13,26]. Moreover, Mendes et al. [13] found no significant differences in chest wall volume among SMI, FIS, and VIS. Therefore, CSMI can effectively stimulate IEIM activity comparable to FIS and VIS, while reducing SCM and SEIM activation.

### Cardiovascular function

This study investigated SV, the amount of blood ejected per heartbeat, during breathing training with FIS and VIS devices. SV significantly decreased during the third inhalation compared to CSMI. This finding aligns with Russo et al. [28] who described the physiological effects of slow breathing in healthy individuals. Respiratory rate influences the harmonics of the blood pressure pulse, which is associated with peripheral vascular resistance, compliance of the aorta, and venous return to the heart. Slow breathing at 6 breaths per minute, characteristic of diaphragmatic breathing, increases venous return through diaphragmatic movement, which compresses the aorta, inferior vena cava, and support cardiac filing. Therefore, venous return increases, leading to greater cardiac preload and, consequently, higher SV [28]. These physiological mechanisms explain our findings that SV was lower during FIS and VIS than during CSMI training.

The heart rate variable showed no significant differences among the FIS, VIS, and CSMI groups. These findings align with that of Zerang et al. in 2022 [11], who reported no statistically significant difference in heart rate when comparing breathing training with an incentive spirometer with deep breathing exercises, a method similar to CSMI training.

CO during FIS breathing training on the 3^rd^, 6^th^, and 9^th^ inhalations were significantly lower than during CSMI. This may be due to greater respiratory muscle engagement during FIS and VIS compared to CSMI, reflecting the interaction between respiratory effort and cardiovascular function. Convertino et al. (2019) [29] described the heart-lung relationship during spontaneous breathing, the diaphragm and external intercostal muscles contract then expanding the chest and causing the pleural lining to stretch, which increases lung volume. However, during positive-pressure inhalation, intrathoracic pressure rises, reducing venous return and right ventricular preload while simultaneously increasing right ventricular afterload through compression of alveolar vessels. These effects collectively reduce the right ventricular SV, and CO, consequently. Therefore, FIS likely resulted in lower CO than VIS and CSMI. In contrast, during CSMI, it was observed that CO remained stable, with only small changes observed in both SV and HR compared to baseline.

### Clinical implications

The findings offer preliminary insights that may inform clinical practice. FIS was associated with greater activation of accessory respiratory muscles and more pronounced changes in cardiovascular function parameters compared to VIS and CSMI. Clinicians should exercise caution when prescribing FIS to individuals with unstable cardiovascular function or those prone to breathlessness. In contrast, VIS and CSMI elicited increased activation IEIM with comparatively stable cardiovascular responses. These techniques may be more suitable for promoting respiratory muscle engagement while minimizing physiological stress. However, as these results are based on healthy participants, further research involving clinical populations is warranted to validate the applicability and safety of these interventions in therapeutic settings.

### Limitations of this study

This study had a few limitations. First, the main inspiratory muscle, the diaphragm, was not monitored due to deep muscle and invasive technique required, which may have limited the study’s ability to characterize respiratory mechanics fully. Second, this study measured respiratory muscle activity only at specific intervals (3^rd^, 6^th^, and 9^th^ breathing cycles). A longer observation period may provide a better understanding of changes in muscle activity over extended exercise durations.

## Conclusion

All breathing types can activate the inferior external intercostal muscles. However, VIS and FIS stimulated greater accessory muscle activity than CSMI. Moreover, FIS resulted in the lowest cardiac output compared to VIS and CSMI. Therefore, VIS and CSMI should be performed to increase IEIM and avoid stimulation of the accessory muscles and changes in cardiovascular function.

## Supporting information

S1 File Dataset of respiratory muscle activities and cardiovascular function variables. (XLSX)
